# Mutations in *MT-ATP6* are a frequent cause of adult-onset spinocerebellar ataxia

**DOI:** 10.1007/s00415-021-10607-5

**Published:** 2021-05-26

**Authors:** Dagmar Nolte, Jun-Suk Kang, Amrei Hofmann, Eva Schwaab, Heidrun H. Krämer, Ulrich Müller

**Affiliations:** 1 Institut für Humangenetik , Justus-Liebig-Universität Giessen, Schlangenzahl 14, Giessen, 35392 Germany; 2 Klinikum der Johann Wolfgang Goethe-Universität, Klinik für Neurologie, Frankfurt, Germany; 3 Praxis für Humangenetik , Wiesbaden, Germany; 4 Klinik für Neurologie , Justus-Liebig-Universität Giessen, Giessen, Germany; 5 Present Address: Neuropraxis, Frankfurt, Germany; 6 Present Address: Klinikum Worms, Klinik für Pädiatrie, Worms, Germany

**Keywords:** Adult-onset ataxia, *MT-ATP6*, ATP synthase, Complex V defect

## Abstract

Adult-onset ataxias are a genetically and clinically heterogeneous group of movement disorders. In addition to nuclear gene mutations, sequence changes have also been described in the mitochondrial genome. Here, we present findings of mutation analysis of the mitochondrial gene *MT-ATP6*. We analyzed 94 patients with adult-onset spinocerebellar ataxia (SCA), including 34 sporadic cases. In all patients, common sequence changes found in SCAs such as repeat expansions and point mutations had been excluded previously. We found pathogenic *MT-ATP* variants in five of these patients (5.32%), two of whom were sporadic. Four of the five mutations have not previously been described in ataxias. All but one of these mutations affect transmembrane helices of subunit-α of ATP synthase. Two mutations (p.G16S, and p.P18S) disrupt transmembrane helix 1 (TMH1), one mutation (p.G167D) affects TMH5, and another one (p.L217P) TMH6. The fifth mutation (p.T96A) describes an amino acid change in close proximity to transmembrane helix 3 (TMH3). The level of heteroplasmy was either complete or very high ranging from 87 to 99%. The high prevalence of pathogenic *MT-ATP6* variants suggests that analysis of this gene should be included in the routine workup of both hereditary and sporadic ataxias.

**Supplementary Information:**

The online version contains supplementary material available at 10.1007/s00415-021-10607-5.

## Introduction

Hereditary adult-onset ataxias are a phenotypically and genetically heterogeneous group of movement disorders. They can be transmitted as autosomal-dominant, autosomal-recessive, X-linked, or mitochondrial traits. Autosomal-dominant spinocerebellar ataxias (SCA) are characterized by gait and limb ataxia, associated with dysarthria and abnormal eye movements in most patients. Additional signs and symptoms may comprise aberrant reflexes, seizures, dystonia, tremor, myoclonus, and cognitive impairment. Mutations have been described in various genes in SCAs. The types of mutations observed are repeat expansions, point mutations, deletions, and insertions in nuclear genes [1]. No obvious genotype/phenotype correlations can be established in most cases. Exceptions include SCA7 characterized by ataxia concurring with retinopathy, and SCA34 that frequently presents with erythrokeratodermia in addition to ataxia [1].

Mutations of mitochondrial DNA frequently underlie ataxia-associated syndromes, even if ataxia is not the major sign [2–4]. One of the genes affected, mitochondrial ATP synthase 6 (*MT-ATP6),* codes for ATP synthase subunit-α which is a subunit of the F_1_F_0_ATP-synthase complex responsible for mitochondrial energy production [5].

*MT-ATP6* mutations including point mutations, deletions and truncations have also been described in adult-onset ataxia patients. The phenotype of ataxia caused by mutations in *MT-ATP6* can frequently not be distinguished from ataxias caused by nuclear gene mutations [6]. In other cases, however, ataxia is associated with various symptoms such as combinations of ataxia with spastic paraplegia [7], motor neuron disease [8], neuropathy [9], myeloneuropathy [10], white matter abnormalities, kidney disease and cognitive decline [11], peripheral neuropathy, diabetes and hypergonadotropic hypogonadism [12], and episodic weakness combined with inherited axonal neuropathy [13]. Of these syndromes, only the complex ataxia-related syndrome described by Kytövouri is caused by a unique mutation of *MT-ATP6*, m.8561C>G (p.P12S) [12], which was formerly not associated with maternally inherited Leigh syndrome (MILS), or neuropathy, ataxia, and retinitis pigmentosa (NARP) syndrome [3].

The degree of heteroplasmy of the mutated gene *MT-ATP6* facilitates classification of some mitochondrial syndromes. Thus, a mutation load of > 90% is frequently found in MILS syndrome [3, 14] and *MT-ATP6* mutations in 70–90% of mitochondrial DNA often cause NARP syndrome [2, 3, 15, 16].

The following study was performed to determine the relative frequency and possible specificity of *MT-ATP6* mutations in patients clinically classified as adult-onset spinocerebellar ataxia.

## Patients and methods

### Genetic analysis

Ninety-four unrelated spinocerebellar ataxia patients were tested for mutations in *MT-ATP6* (ENSG00000198899). Eighty-six patients were of German origin, three were Russians, two Polish, and one patient each came from Turkey, Spain, and Italy. The study was approved by the Ethics Committee of the University of Giessen. Patients gave written informed consent according to the guidelines of the German Genetics Diagnostics Act. All patients were examined and diagnosed at specialized German movement disorder centers. Other causes of ataxic movement disorders such as neoplasia, stroke, CNS infection, multiple sclerosis, vitamin deficiency, and alcohol abuse were excluded in all patients. Sixty patients had a positive family history consistent with autosomal-dominant or mitochondrial inheritance. Thirty-four patients were classified as sporadic.

DNA was extracted from peripheral blood. Repeat expansions at loci SCA1-3, SCA6-8, SCA10, SCA12, and SCA17 were excluded. Similarly, no pathogenic variants were detected at loci SCA11 (*TTBK2*), SCA13 (*KCNC3*), SCA14 (*PRKCG*), SCA19 (*KCND3*), SCA23 (*PDYN*), SCA27 (*FGF14*), SCA28 (*AGF3L2*), and SCA38 (*ELOVL5*). Large deletions at SCA15/16 (*ITPR1/SUMF1*) were excluded by quantitative PCR.

A 953-bp fragment of *MT-ATP6* was amplified by PCR using primers mtATP6_F: 5′-GCCCACCATAATTACCCC-3′, and mtATP6_R: 5′-GCCTAGTATGAGGAGCGTTATG-3′. PCR fragments were sequenced in both directions.

### Analysis of degree of heteroplasmy

Heteroplasmy levels for m.8572G > A (p.G16S), m.8578C > T (p.P18S), m.8812A > G (p.T96A), m.9026G > A (p.G167D), and m.9176 T > C (p.L217P) were determined by pyrosequencing as described earlier [6]. DNA of the five patients was amplified by PCR to generate short products. One of the primers was biotinylated to facilitate isolation of the template strand via streptavidin. For pyrosequencing, a sequencing primer was used in close proximity to the mutation. Pyromark Assay Design Software v.2.0 (Qiagen/Hilden) was used for design of the variant-specific assays. Pyrosequencing was done on a Pyromark Q24 sequencer according to the manufacturer’s instructions. Assays were repeated at least twice.

A single PCR product was generated for closely adjacent variants m.8572G > A (p.G16S) and m.8578C > T (p.P18S). Primers were Pyro_G16S_P18S_F: 5′-TCTGTTCGCTTCATTCATTGC-3′ and 5′-biotinylated reverse primer Pyro_G16S_P18S_R: 5′-GAGGGGGAAATAGAATGATCAGTA-3′. Both variants were quantified in DNA of patient #960 (m.8572G > A), or patient #982 (m.8578C > T) using primer PyroSeq_G16_P18_F: 5′-TGCCCCCACAATCCT-3′.

Variant m.8812A > G (p.T96A) was analyzed with 5′-biotinylated forward primer Pyro_T96A_F: 5′-CTCGGACTCCTGCCTCACT-3′, and Pyro_T96A_R: 5′-CTGTGCCCGCTCATAAGG-3′. Reverse primer used for quantification was PyroSeq_T96A_R: 5′-GGCTAGGTTTATAGATAGTT-3′.

Primers for variant m.9026G > A (p.G167D) were Pyro_G167D_F: 5′-AACCAATAGCCCTGGCCGTAC-3′ and 5′-biotinylated Pyro_G167D_R: 5′-CGCTTCCAATTAGGTGCATGA-3′. Primer used for quantification was PyroSeq_G167D_F: 5′-CTAACCGCTAACATTACTG-3′.

Variant m.9176T > C (p.L217P) was analyzed using primers Pyro_L217P_F: 5′- TCGCCTTAATCCAAGCCTAC-3′, and 5′-biotinylated Pyro_L217P_R: 5′- ATTATGTGTTGTCGTGCAGGTAGA-3′. Quantification was performed with primer PyroSeq_L217P_F: 5′-CCTACGTTTTCACACTTC-3′.

### Prediction of pathogenicity

Pathogenicity of observed variants was analyzed in silico (Table 1). Programs used were MutationTaster2 (http://www.mutationtaster.org) [17], Polyphen-2 (http://genetics.bwh.harvard.edu/pph2/) [18], PROVEAN (http://provean.jcvi.org) [19], SIFT (https://sift.bii.a-star.edu.sg) [20, 21], and Variant Effect Predictor (VEP, https://www.ensembl.org/info/docs/tools/vep/index.html) [22], which is a modified version of a combination of SIFT and Polyphen-2 prediction programs.

**Table 1:** Prediction, MitoMap frequency, and ACMG classification of *MT-ATP6* missense variants detected in a cohort of 94 SCA patients

mDNA/cDNA/protein change/ cases in cohort	SNV number MAF (ALFA database)	MutationTaster (Score)	Polyphen-2 (Score)	PROVEAN (Score)	SIFT (Score)	VEP/ Ensembl SIFT / Polyphen (Score)		MitoMap frequency	ACMG Classification criteria
m.8572G > A c.46 G > A, p.G16S, 1	rs28502681 A = 0.0009	Disease causing (0.9961)	Probably damaging (0.895)	Deleterious (− 4.623)	not tolerated (0.02)	0.03	0.498	0.344%	Class 3 (variant of uncertain significance) PM1, PP3
m.8578C > T c.52C > T, p.P18S, 1	rs1556423492 T = 0.0004	Polymorphism (0.9517)	Probably damaging (0,999)	Deleterious (− 6.594)	Tolerated (0.17)	0.03	0.996	0.058%	Class 4 (likely pathogenic) PS4, PM1, PP3
m.8584G > A c.58G > A, p.A20T, 2	rs3135028 A = 0.0067	Polymorphism (0.9999)	Benign (0.004)	Neutral (− 0.404)	Tolerated (0.21)	0.24	0.012	5.558%	Class 1 (benign) BA1
m.8701A > G c.175A > G, p.T59A, 3	rs2000975 G = 0.06433	Polymorphism (0.9999)	Benign (0.002)	Neutral (− 0.935)	Tolerated (0.66)	0.51	0.005	32.975%	Class 1 (benign) BA1
m.8705 T > C c.179 T > C, p.M60T, 2	rs878959404 C = 0.0043	Polymorphism (0,9999)	benign (0.000)	Neutral (0.320)	tolerated (0.30)	0.68	0.0	0.383%	Class 2 (likely benign) BP4, BP6
m.8723G > A c.197G > A, p.R66Q, 1	rs unknown	Polymorphism (0.9997)	Benign (0.021)	Neutral (− 0.523)	Tolerated (0.51)	0.55	0.012	0.159%	Class 2 (likely benign) BP4, BP6
m.8764G > A c.238G > A, p.A80T 1	rs1556423534 A = 0.0018	Polymorphism (0.9999)	Benign (0.001)	Neutral (− 1.221)	Tolerated (0.26)	0.12	0.007	0.207%	Class 2 (likely benign) BP4, BP6
m.8812A > G c.286A > G, p.T96A, 1	rs1556423543 G = 0.0018	Polymorphism (0.9266)	Probably damaging (0.994)	Deleterious (− 3.891)	Not tolerated (0.03)	0.05	0.988	0.118%	Class 3 (variant of uncertain significance) PM1, PP3
m.8950G > A c.424G > A, p.V142I, 1	rs1556423574 A = 0.0008	Polymorphism (0.9999)	Benign (0.0)	Neutral (0.118)	Tolerated (1.0)	1.0	0.0	0.151%	Class 2 (likely benign) BP4, BP6
m.9026G > A c.500G > A, p.G167D, 1	COSV62293160	Disease causing (0.9999)	Probably damaging (1.0)	Deleterious (− 6.275)	Not tolerated (0.00)	0.0	0.999	0.006%	Class 5 (pathogenic) PS1, PM1, PM2, PM5
m.9055G > A c.529G > A, p.A177T, 7	rs193303045 A = 0.1556	Polymorphism (0.9988)	Probably damaging (0.845)	Deleterious (− 2.606)	Tolerated (0.16)	0.1	0.399	4.244%	Class 1 (benign) BS1, BS4
m.9067A > G c.541A > G, p.M181V, 2	rs unknown	Polymorphism (0.9999)	Benign (0.003)	Neutral (− 0.967)	Not tolerated (0.01)	0.01	0.007	0.070%	Class 2 (likely benign) BP4, BP6
m.9070 T > G c.544 T > G, p.S182A, 1	rs879190502 G = 0.0020	Polymorphism (0.9999)	Benign (0.225)	Neutral (− 0.122)	Tolerated (0.57)	0.12	0.182	0.126%	Class 2 (likely benign) BP4, BP6
m.9176 T *C* c.650 T> C, p.L217P 1	rs199476135 C = MAF unknown	Disease causing (0.9999)	Probably damaging (0.999)	Deleterious (− 6.258)	Not tolerated (0.00)	0.0	0.998	0.006%	Class 5 (pathogenic) PS1, PS3, PS4

*SCA* spinocerebellar ataxia, *MAF* minor allele frequency, underline: likely pathogenic variants; ALFA database: (https://www.ncbi.nlm.nih.gov/snp/docs/gsr/alfa) [36]; ACMG classification, detailed information is given in [24]

Frequency of each variant detected (Table 1) was analyzed by searching the database MitoMap (https://www.mitomap.org) [23]. Variants were classified according to the ACMG guidelines [24] (Table 1).

### Clinical findings

#### Patient 1

**Fig. 1:**
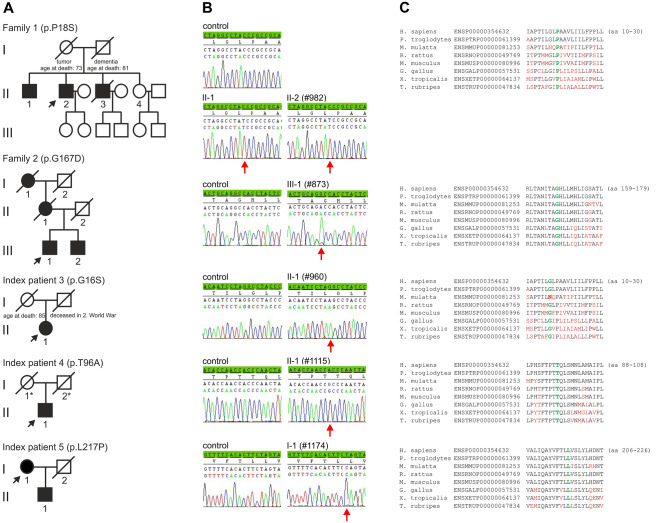
**a** Pedigrees of the German adult-onset SCA cases. Black symbols indicate affected probands. Index patients are marked by arrow. Probands for whom no clinical information was available are highlighted by an asterisk. **b** Electropherograms of sequences of index patients and controls. The relevant base changes are indicated by arrow. **c** Amino acid sequence alignments of ATP synthase subunit-α orthologs. Name of species and protein identifier numbers are given on the left. Amino acids mutated in patients are evolutionarily highly conserved and are highlighted in green. Non-conserved amino acid residues are given in red. H., Homo; P., Pan; M., Macaca; R., Rattus; M., Mus; G., Gallus; X., Xenopus; T., Takifugu

Disease onset in male patient 1 (#982, family 1, II-2, Fig. 1a, Table 2) was at age 53 when he presented with gait instability and frequent falls. At age 56, comprehensive neurological examination revealed mild and slowly progressive gait ataxia, postural instability, dysdiadochokinesia, moderate horizontal nystagmus and mild dysarthria. Fine motor movements were not impaired. While psychiatric symptoms were excluded, the patient complained of moderate lack of concentration and forgetfulness. His older brother who had perinatal asphyxia presented with generalized dystonia and mild ataxic gait. His younger brother died at age 50 of unknown causes. However, a psychiatric disorder and tremor had been excluded. The patient´s sister was healthy at her last examination at age 48.

**Table 2:** Mutations in *MT-ATP6* associated with adult-onset ataxia, prediction analysis, and clinical features

Family/patient (sex, current age, identifier)	mtDNA/cDNA change	Deduced aa change/localization	Prediction Polyphen2, PROVEAN, SIFT (Score)	Age at onset/first symptoms	Age at examination/clinical symptoms	MRI
*Family 1*						
II-1 (M, 65)	m.8578C > T; c.52C > T	p.P18S TMH1	Prob. damaging (0.999), deleterious (-6.594), tolerated (0.17)	Umbilical cord prolapse, 45, dystonia	58, generalized dystonia, dystonic dysarthria, dysphagia, mild psychomotor developmental delay	Hyperintensity of bilateral putamen and precentral gyrus
II-2 (M, 63) index patient (#982)	m.8578C > T; c.52C > T	p.P18S TMH1	Prob. damaging (0.999), deleterious (-6.594), tolerated (0.17)	53, gait abnormality, frequent falls	56, mild and slowly progressive gait ataxia, horizontal nystagmus	Cerebellar atrophy
II-3 (M, deceased)	n.d			n.i	obsessive–compulsive disorder, imbalance problems, deceased at 51	n.d
II-4 (F, 55)	m.8578C > T; c.52C > T	p.P18S TMH1	Prob. damaging (0.999), deleterious (-6.594), tolerated (0.17)	Asymptomatic	48, (last examination), asymptomatic	n.d
*Family 2*						
I-1 (F, deceased)	n.d			50, gait abnormality		n.d
II-1 (F, deceased)	n.d			54, head and hand tremor		n.d
III-1 (M, 73) index patient (#873)	m.9026G > A; c.500G > A	p.G167D TMH5	Prob. damaging (1.0), deleterious (-6.275), not tolerated (0.00)	46, progressive head and hand tremor	62, mild gait ataxia, undirected falls, tremor with lateral shift to the right and rotation to the left side, dysmetric finger-to-nose and knee–heel test, lifted saccades, mild cognitive impairment	Colliquative necrosis of the temporal lobe
*Index patient 3* (F, 85) (#960)	m.8572G > A c.46G > A;	p.G16S TMH1	Prob. damaging (0.895), deleterious (-4.623), not tolerated (0.02)	65, mild gait ataxia	75, mild but progressive gait ataxia, dysmetria, dysarthria, restless-legs-syndrome	Global brain atrophy, incl. Cerebellum
*Index patient 4* (M, 61) (#1115)	m.8812A > G c.286A > G;	p.T96A adjacent to TMH3 (aa 97–117)	Prob. damaging (0.994), deleterious (-3.891), not tolerated (0.03)	46, gait abnormality	56, progressive gait ataxia	Cerebellar atrophy
*Index patient 5* (F, 79) (#1174)	m.9176T> C; c.650T > C	p.L217P TMH6	Prob. damaging (0.999), deleterious (-6.258), not tolerated (0.00)	46, frequent falls, gait insecurity	75, severe progressive gait ataxia, cerebellar dysarthria, saccadic gaze	Moderate cerebellar atrophy

*F* female, *M* male, *aa* amino acid, *m.* mitochondrial genomic DNA, *TMH* transmembrane helix domain, *n.d.* not done/unavailable for testing; n.i.: no information

#### Patient 2

Male patient 2 (#873, family 2, III-1, Fig. 1a, Table 2) came to clinical attention at age 62 because of ataxic gait, frequent falls, and tremor. Dysmetria was diagnosed by finger-to-nose and knee-heel test. The patient reported first occurrence of postural and action tremor of the hands at age 46. At the time of investigation, lifted saccades and abnormal executive function were diagnosed. Brain MRI revealed a colliquative necrosis of the temporal lobe. At age 73, ataxic wide-based gait had worsened. Tremor that was initially confined to the hands, now also affected the head and had become the major sign. SCA-loci that are associated with tremor (SCA12, and SCA15/16) have been excluded in this patient. The patient´s younger brother, his deceased mother, and maternal grandmother had had similar signs and symptoms, of which tremor and mild ataxic gait were most striking.

#### Patient 3

Female patient 3 (#960, II-1, Fig. 1a, Table 2) was sporadic with none of her parents affected. In the patient, a mild spinocerebellar ataxia was diagnosed at age 65. The ataxia was progressive but did not affect the ability to walk without a cane for at least short distances at age 75.

#### Patient 4

At age 56, sporadic male patient 4 (#1115, II-1, Fig. 1a, Table 2) came to clinical attention due to a pure, progressive ataxic syndrome. MRI revealed distinct cerebellar atrophy. No health problems, in particular no movement disorders have been reported in his parents. His mother died at age 85. His father was killed in World War II.

#### Patient 5

Abnormal gait and frequent falls first occurred in female patient 5 (#1174, I-1, Fig. 1a, Table 2) at age 46. At age 75, a comprehensive neurological examination revealed pronounced dysarthria and a saccadic gaze sequence. Performance of directed movements and abnormal gait had severely worsened. Walking distance was only a few meters even when using a walker. MRI revealed distinct cerebellar atrophy. Her son suffered from similar symptoms that were diagnosed in his thirties.

## Results and discussion

In 94 adult-onset SCA cases, we detected 14 variants of *MT-ATP6* that result in non-synonymous amino acid (aa) changes (Table 1). Five of these variants were predicted to be deleterious by at least three of the five in silico tools applied (Table 1). These variants are m.8572G > A (c.46G > A; p.G16S) detected in sporadic patient 3 (II-1, Table 2, Fig. 1b), m.8578C > T (c.52C > T; p.P18S) (patients II-1 and II-2 of family 1, Table 2, Fig. 1b), m.8812A > G (c.286A > G; p.T96A) (sporadic patient 4, II-1, Table 2, Fig. 1b), m.9026G > A (c.500G > A; p.G167D) (family 2, patient III-1, Table 2, Fig. 1b), and m.9176T > C (c.650T > C; p.L217P) (patient 5, I-1, Table 2, Fig. 1b). An additional variant, m.9055G > A (c.529G > A; p.A177T), was classified as deleterious by two programs, but could be excluded, because it occurred multiple times in our collective and is also frequent in controls as reflected by the high MitoMap frequency of 4.24% (Table 1).

All deleterious variants but variant m.9176T > C (c.650T > C; p.L217P) have not been associated with mitochondrial disease before. These variants were classified as class 5/pathogenic (m.9026G > A, m.9176T > C), class 4/likely pathogenic (m.8578C > T), and class 3/variant of uncertain significance (m.8572G > A, m.8812A > G) according to the ACMG guidelines [24]. Pathogenicity of these variants is further supported by phylogenetic conservation of the affected aa residues (Fig. 1c), a finding that indicates an important role of these aa’s in normal protein function.

Of the aa changes observed, all but one affect the helix structure of transmembrane domains of subunit-α of ATP synthase.

The two most proximal variants were detected in sporadic patient 3 (II-1), and in patients II-1, and II-2 of family 1. Of these, m.8572G > A (c.46G > A) results in a glycine to serine change at aa position 16 (p.G16S). The mutation m.8578C > T (c.52C > T) of family 1 is located adjacent to m.8572 and results in the substitution of a proline by a serine at aa position 18 (p.P18S). The pyrograms revealed homoplasmy for both m.8572G > A (p.G16S), and m.8578C > T (p.P18S) (Suppl. Figure 1). Both mutations affect the first transmembrane helix (TMH1) of subunit-α of ATP synthase and appear to disturb proton translocation. However, most disease-causing alterations of ATP synthase subunit-α appear to be located in the three distal transmembrane helices (TMH4-6) independent of the patient´s phenotype [4, 13, 25, 26].

The mutation m.8561C > G (p.P12R) of subunit-α in a patient with adult-onset ataxia, neuropathy, diabetes, and hypergonadotropic hypogonadism was shown to interfere with assembly of complex V of the mitochondrial respiratory chain by the alteration of two ATP synthase subunits. This results in impaired ATP synthesis [12].

In sporadic patient 4 (II-1), two non-synonymous aa changes were detected. Variant m.8723G > A (c.197G > A; p.R66Q) was predicted to be likely benign (Table 1). In contrast, variant m.8812A > G (c.286A > G) shows a mutation load of 97% (Suppl. Figure 1) and results in replacement of threonine by alanine at aa position 96 (p.T96A). This variant is located adjacent to TMH3, which spans aa 97–117 as shown in UniProtKB (http://www.uniprot.org/uniprot/P00846).

Two variants were found in patient III-1 of family 2. Of these sequence changes, m.8950G > A (c.424G > A; p.V142I) was classified as likely benign (Table 1). In contrast, m.9026G > A (c.500G > A) is predicted to be pathogenic (Table 1). This mutation has a heteroplasmic load of about 87% (Suppl. Figure 1) and results in the replacement of a glycine by an aspartate at aa position 167 (p.G167D) of TMH5. A previous finding of an aa change at the same position (p.G167S) in patients with NARP-MILS syndrome [27] supports a possible impairment of the ATP synthase subunit α. Recently, m.9026G > A was also described in a child with intellectual disability, headaches, myalgias, and fatigue. However, a low mutation load of 16–23% in various tissues makes a correlation with the child's symptoms difficult [26].

Other deleterious aa changes associated with reduced ATP synthase activity have been described in close proximity to p.G167D. Among these, p.L170P was described in patients with cognitive delay, and early-onset ataxia [28]. p.L170P was also the first *MT-ATP6* mutation associated with pure adult-onset ataxia [6]. Both our patient III-1 of family 2 carrying the p.G167D mutation and the patient described by Pfeffer [6] did not have cerebellar atrophy. In contrast to Pfeffer´s and Sikorska´s cases, the patient described here displayed a severe dystonic tremor. This finding shows that—similar to autosomal-dominant ataxia cases [1]—a strict genotype–phenotype correlation can also not be established in mitochondrial ataxia [3, 26, 29, 30].

Variant m.9176T > C (c.650T > C) was almost homoplasmic with a 99% mutation load (Suppl. Figure 1) in patient 5 (I-1). The deduced amino acid change of leucine to proline at position 217 (p.L217P) is located in TMH6. Unlike the novel mutations described above, m.9176T > C has been reported at least 30 times in several disorders with highly variable disease duration and age of onset [4, 23, 30, 31]. These disorders include a late-onset hereditary spastic paraplegia-like syndrome [7], MILS [32, 33], and ataxia in combination with familial bilateral striatal necrosis [34].

The five pathogenic variants of *MT-ATP6* described here result in a prevalence of 5.32% in our group of adult-onset SCA patients. Two of these mutations occurred in the 34 patients with negative family histories, this amounts to 5.88% that is even higher than the overall prevalence in the cohort. The overall prevalence of 5.32% is significantly higher than the 3.13%, that were reported by Pfeffer et al. in a study of 64 ataxia cases [6]. Our findings are in agreement with Pulkes’ conjecture [35] of an important role and comparatively frequent occurrence of *MT-ATP6* mutations in adult-onset ataxia patients.

In conclusion, *MT-ATP6* mutations mainly affect the transmembrane helical domains of subunit-α of ATP synthase. Given the relatively frequent finding of *MT-ATP6* mutations in SCA patients, this gene should be routinely analyzed in SCA patients, even in the absence of positive family history, once repeat expansions have been excluded.

## Supplementary Information

Below is the link to the electronic supplementary material.

**Supplementary file 1** (PDF 209 KB)
