# Supplementary material for: Mutations in MT-ATP6 are a frequent cause of adult-onset spinocerebellar ataxia
Source: J Neurol. 2021 May 26;268(12):4866–73. doi: 10.1007/s00415-021-10607-5 (PMC8563540; doi:10.1007/s00415-021-10607-5)
Supplement: Supplementary file 1 — (PDF 209 KB) [file 415_2021_10607_MOESM1_ESM.pdf]

Supplemental Fig. 1

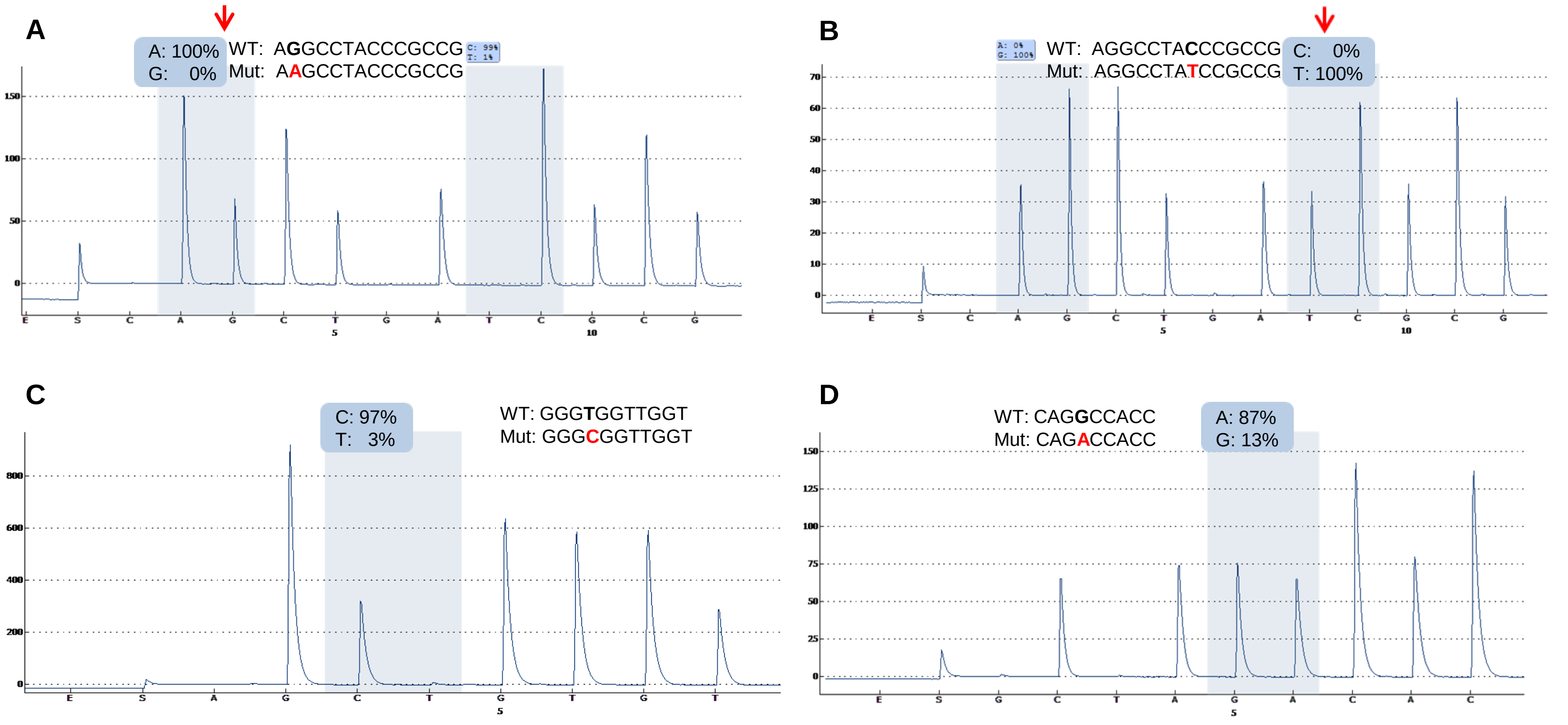

E

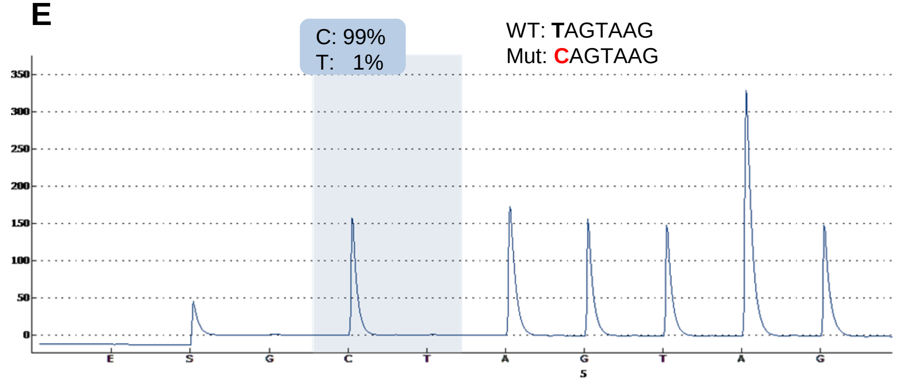

Suppl. Fig. 1. Detection of heteroplasmy levels with pyrosequencing assays. Y-axis: relative light intensity, X-axis: dispensation of nucleotides. Pyrograms, heteroplasmy degrees, and information on WT- and Mut-sequence are given. Please note that in (C) the forward strand was sequenced, therefore, reverse sequence is given. A: Patient #960, m.8572G>A (p.G16S); B: Patient #982, m.8578C>T (p.P18S); C: Patient #1115, m.8812A>G (p.T96A); D: Patient #873, m.9026G>A (p.G167D); E: Patient #1174, m.9176T>C (p.L217P). WT: wild type sequence, Mut: sequence with mutant allele. Analysed region is marked by a red arrow.
